# Comprehensive Evaluation of Five Pecan (*Carya illinoinensis*) Cultivars at Seedling Stage Based on Principal Component Analysis

**DOI:** 10.3390/plants14172705

**Published:** 2025-08-30

**Authors:** Jiaju Zhu, Juan Zhao, Longtao Lu, Pengpeng Tan, Kaikai Zhu, Fangren Peng

**Affiliations:** 1 National Key Laboratory for the Development and Utilization of Forest Food Resources, Nanjing Forestry University, Nanjing 210037, China; zjj010802@163.com (J.Z.); cxyl@njfu.edu.cn (J.Z.); l2505841171@163.com (L.L.); tanpengpeng2002@163.com (P.T.); kkzhu@njfu.edu.cn (K.Z.); 2 College of Forestry, Nanjing Forestry University, Nanjing 210037, China

**Keywords:** pecan, growth traits, anatomical structure, photosynthesis, principal component analysis, comprehensive evaluation

## Abstract

Pecan (*Carya illinoinensis* [Wangenh]. K. Koch) germplasm resources are abundant, yet the characteristics of each cultivar at the seedling stage remain insufficiently understood. This study systematically evaluated the growth parameters, photosynthetic traits, and anatomical structures of one-year-old grafted seedlings from five pecan cultivars: “Pawnee”, “Mandan”, “Nacono”, “Caddo”, and “Creek”. Principal component analysis (PCA) was employed to comprehensively assess 56 indicators. The results indicated that, in terms of vegetative growth, leaf area and biomass of “Nacono” and “Creek” were significantly greater than those of “Pawnee” (*p* < 0.05). “Mandan” ranked second. Additionally, the seedling quality index of “Creek” was markedly superior to all other cultivars (*p* < 0.05). Anatomically, “Pawnee” exhibited greater leaf thickness, more highly differentiated palisade tissue, and the development of the main vein. By contrast, “Mandan” displayed larger branch radius, cortex thickness, and pith radius, accompanied by finer vessels and large but sparsely distributed stomata (*p* < 0.05). Regarding photosynthetic performance, “Mandan” accumulated the highest concentrations of photosynthetic pigments and achieved the greatest photosynthetic efficiency, significantly outperforming the remaining cultivars (*p* < 0.05). The PCA-based comprehensive evaluation revealed that “Mandan” outperformed the other cultivars in seedling growth, making it the most suitable for promotion, followed by “Creek”, “Nacono”, “Caddo”, and “Pawnee”. This research offers a theoretical foundation for the breeding, promotion, and application of superior pecan cultivars.

## 1. Introduction

Pecan (*Carya illinoinensis* (Wangenh.) K. Koch) is a species within the genus *Carya*, belonging to the Juglandaceae family [1]. It is characterized by its large size, thin shell, and high kernel yield. The kernels are prized for their excellent taste, aroma, and absence of astringency. Additionally, pecans are rich in amino acids, vitamins, trace elements, and other nutrients beneficial to human health [2]. With an oil content reaching up to 70% and an unsaturated fatty acid content of 97%, pecans exhibit significant antioxidant properties, making them an important woody oil crop [3]. Pecan extracts also show potential in inhibiting cancer cell proliferation [4]. The tree has a tall, upright form, attractive shape, and fine wood grain, making it valuable both as timber and in landscaping [5]. Native to the United States and northern Mexico [6], the pecan is one of the most important and valuable nut tree species in North America [7]. Global pecan (*Carya illinoinensis*) production is currently estimated at 700–800 million pounds of in-shell nuts, with the United States and Mexico accounting for over 90% of the total, but over the past decade, South Africa has emerged as a major producer of pecan nuts [8]. Since its introduction in the 18th century, pecans have been widely cultivated in over 20 countries, including the United States, Mexico, Brazil, and China. In China, pecans are now grown in 22 provinces, including Anhui, Yunnan, Jiangsu, and Zhejiang [9,10]. In pecan breeding, seedlings generally require 10 to 15 years to reach fruit-bearing maturity, and their yields are typically low and unstable. Consequently, seedlings are primarily utilized as rootstocks. Pecan propagation is commonly carried out through branch grafting or square bud grafting. Grafted trees usually initiate flowering in the second or third year following planting and begin fruiting in the fourth or five year, thereby significantly reducing the juvenile phase and accelerating productivity [11].

However, the selection of superior cultivars remains a critical factor limiting the development of the pecan industry [1]. Additionally, substantial differences exist in the growth stress resistance and the suitability of growing areas among the introduced cultivars [12]. Growth indicators and the anatomical structure of leaves are vital for assessing plant adaptability and play a crucial role in understanding the physiological and ecological responses of plants to environmental conditions [13,14]. Plant leaves are highly sensitive to environmental factors [15]. Previous studies have demonstrated that leaf-related indices are closely linked to photosynthetic efficiency and water usage [16]. The anatomical features of leaves and the characteristics of leaf veins influence the dynamic water balance within leaves, thereby affecting the plant’s drought resistance [17]. Several leaf anatomical indicators, such as stomatal density, the thickness of the upper and lower epidermal cells, the thickness of the palisade and spongy tissues, the ratio of palisade to spongy tissue thickness, and the structural tightness or looseness of the leaf, have been shown to influence plant stress resistance and are commonly used as research indicators for plant stress resilience [1,18,19,20]. Furthermore, greater plant biomass is typically associated with enhanced tolerance to adverse environmental conditions [21]. Photosynthesis serves as the foundation for plant growth, and photosynthetic parameters are among the most important indicators for evaluating superior cultivars [22]. Chlorophyll fluorescence provides insights into a range of critical regulatory processes in plants, offering a precise reflection of their growth and developmental status [23]. Given the diverse geographical and climatic conditions across the country, it remains essential to conduct adaptability tests and evaluations on pecan cultivars before promoting them as superior cultivars. Currently, the pecan industry in China faces challenges, including a slow breeding process and a lack of superior cultivars. Integrating morphological, photosynthetic, and anatomical traits at the one-year grafted-seedling stage compresses the traditional 7–10-year pecan cultivar assessment into just a few months. The resulting early-screening data can guide differentiated cultivation strategies for drought-prone, seasonally water-limited, or high-light/temperature regions, thereby markedly reducing both breeding and extension risks. For the first time, this study integrates leaf anatomy, photosynthetic physiology, and morphological traits to systemically differentiate pecan cultivars at the seedling stage via PCA. This approach fills a technological gap in “early-stage, non-destructive, multi-dimensional screening,” offering a practical solution to shorten the juvenile evaluation period and accelerate early identification of superior germplasm.

## 2. Results

### 2.1. Comparison of Growth at Different Stages of Pecan Seedlings

#### 2.1.1. Plant Height, Rootstock Diameter, and Scion Diameter

**Figure 1:**
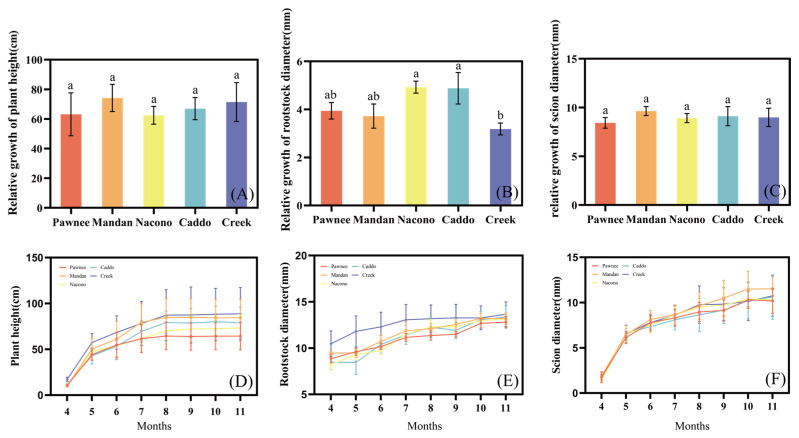
The growth differences and relative growth increment of different cultivars of pecan seedlings at the seedling stage. Relative growth of plant height among different pecan cultivars (**A**). Relative growth of rootstock diameter among different pecan cultivars (**B**). Relative growth of scion diameter among different pecan cultivars (**C**). Differences in plant height over time among different pecan cultivars (**D**). Differences in rootstock diameter over time among different pecan cultivars (**E**). Differences in Scion over time among different pecan cultivars (**F**). Different lowercase letters above the bars indicate the growth differences among different cultivars with *p* < 0.05.

This experiment investigated the growth differences among grafted seedlings of various pecan cultivars at the seedling stage. The results showed that, although numerical differences in relative height and scion diameter increments existed among cultivars, one-way ANOVA indicated no significant differences (*p* > 0.05) (Figure 1A,C). Only the stem-diameter increment of “Creek” was significantly smaller than that of “Nacono” and “Caddo” (*p* < 0.05) (Figure 1B). During the growth period, the growth rate of all cultivars followed a general pattern of rapid growth initially, which slowed down over time, with a noticeable decrease in growth after August. On August, mean plant height ranged from 64.40 cm (“Pawnee”) to 87.19 cm (“Creek”) (Figure 1D); mean rootstock diameter ranged from 11.36 mm (“Pawnee”) to 13.17 mm (“Creek”) (Figure 1E); and mean scion diameter ranged from 8.96 mm (“Pawnee”) to 9.96 mm (“Creek”) (Figure 1F). Height, rootstock diameter, and scion diameter exhibited no significant genotypic differences, possibly due to strong environmental buffering effects during this early growth stage.

#### 2.1.2. Leaf Length, Leaf Width, and Leaf Area

As shown in Table 1, the leaves of “Creek” were significantly longer than those of “Pawnee”, “Nacono”, and “Caddo” (*p* < 0.05). Leaf length of Mandan did not differ significantly from that of the other cultivars (*p* > 0.05). “Nacono” exhibited the widest leaves, significantly broader than those of the other cultivars (*p* < 0.05). The leaf area of both “Creek” and “Nacono” was significantly larger than that of “Pawnee” and “Caddo” (*p* < 0.05). The relatively high leaf length and width of “Pawnee”, “Mandan”, and “Creek” indicate that these leaves are narrower and longer. In contrast, the leaves of “Nacono” and “Caddo” are wider and more rounded, exhibiting an elliptical shape.

#### 2.1.3. Biomass and Quality Index of Seedlings

As shown in Table 2, although numerical differences were observed in leaf biomass and shoot/root ratio among cultivars, neither parameter differed significantly (*p* > 0.05). Mean leaf biomass ranged from 24.85 g (“Pawnee”) to 31.27 g (“Nacono”), whereas the mean root-to-shoot ratio varied from 1.66 (“Caddo”) to 2.13 (“Mandan”). The cultivar with the highest stem biomass was “Creek”, which was significantly greater than that of “Pawnee” (*p* < 0.05). Stem biomass of “Mandan”, “Nacono”, and “Caddo” did not differ significantly from that of the other two cultivars (*p* > 0.05). The root biomass of “Mandan” and “Creek” was significantly higher than that of the other cultivars (*p* < 0.05), whereas “Pawnee” had the smallest root biomass, measuring only 99.98 g. The total biomass of both “Creek” and “Mandan” was significantly higher than that of “Pawnee” (*p* < 0.05), while they did not differ significantly from that of the other two cultivars. (*p* > 0.05). Furthermore, the graftling quality index of “Creek” was significantly higher than that of the other four cultivars (*p* < 0.05), while no significant differences in graftling quality index were observed among the other four cultivars (*p* > 0.05).

### 2.2. Comparison of the Anatomical Structures of Different Pecans at the Seedling Stage

#### 2.2.1. Leaf Anatomical Structure

**Figure 2:**
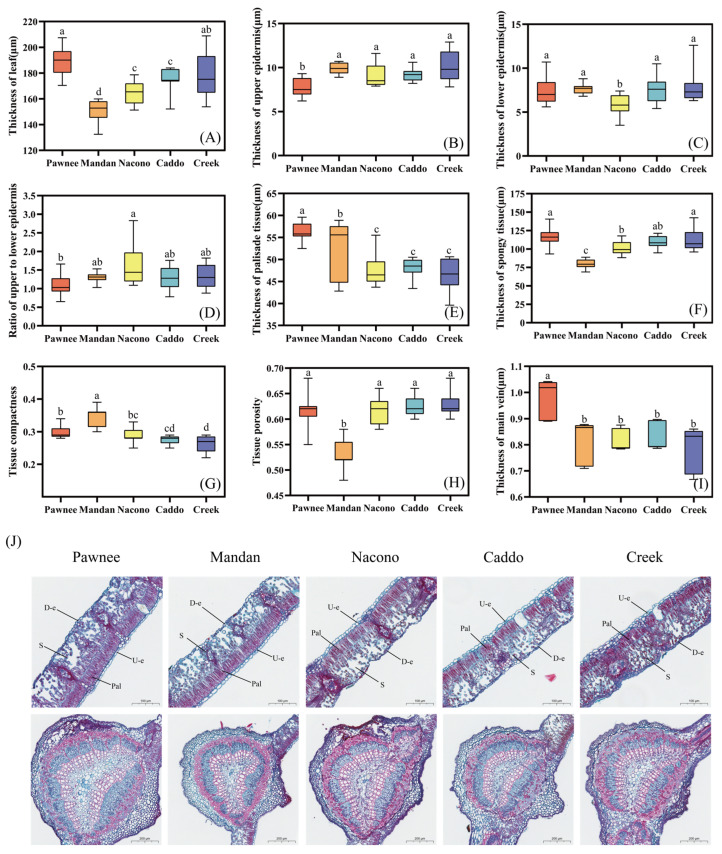
Differences in the leaf anatomical structure of different cultivars of pecan seedlings. Thickness of leaf among different pecan cultivars (**A**). Thickness of upper epidermis among different pecan cultivars (**B**). Thickness of lower epidermis among different pecan cultivars (**C**). Ratio of upper to lower epidermis among different pecan cultivars (**D**). Thickness of Palisade tissue among different pecan cultivars (**E**). Thickness of Spongy tissue among different pecan cultivars (**F**). Tissue compactness among different pecan cultivars (**G**). Tissue porosity among different pecan cultivars (**H**). Thickness of main vein among different pecan cultivars (**I**). Schematic diagram of leaf anatomical structure among different pecan cultivars (**J**). Different lowercase letters above the bars indicate the differences in leaf anatomical structure among different cultivars with *p* < 0.05. U-e, upper epidermis; L-e, lower epidermis; S, spongy tissue; Pal, palisade tissue.

The leaf thickness of the “Pawnee” cultivar was the greatest, being 5.7% thicker than that of “Creek”, with a significant difference (*p* < 0.05). It was also 7.2% thicker than “Caddo”, 13.0% thicker than “Nacono”, and 20.35% thicker than “Mandan”, all of which were extremely significant (*p* < 0.01) (Figure 2A). The upper epidermis of cultivars such as “Creek” and “Mandan” was significantly thicker than that of “Pawnee” (*p* < 0.05), while, except for “Pawnee”, the upper epidermal thicknesses of the other four cultivars showed no significant differences (*p* > 0.05) (Figure 2B). The lower epidermis of “Nacono” was the thinnest (*p* < 0.05), while, except for “Nacono”, the lower epidermal thicknesses of the other four cultivars showed no significant differences (*p* > 0.05) (Figure 2C). The upper to lower epidermis ratio of “Nacono” was significantly greater than that of “Pawnee” (*p* < 0.05), whereas no significant differences were detected among the other four cultivars except for “Pawnee” (*p* > 0.05) (Figure 2D). The thickness of the palisade tissue in “Pawnee” was significantly greater than that in “Mandan” by 7.1% (*p* < 0.05), and extremely significantly higher than in “Nacono”, “Caddo”, and “Creek” by 15.6%, 14.7%, and 17.6%, respectively (*p* < 0.01) (Figure 2E). Additionally, the thickness of the spongy tissue in “Pawnee” was significantly higher than in “Mandan” and “Nacono”, being 31.3% and 13.0% greater, respectively (*p* < 0.05), and no significant differences were observed among the other four cultivars (*p* > 0.05) (Figure 2F). The tissue structure tightness of “Mandan” was significantly higher than that of “Pawnee” and “Nacono” (*p* < 0.05), and extremely significantly higher than that of “Caddo” and “Creek” (*p* < 0.01). Moreover, no significant differences were detected among “Nacono”, “Caddo”, and “Creek” (*p* > 0.05) (Figure 2G). The porosity of the tissue structure in “Mandan” was significantly lower than that in “Nacono”, “Caddo”, and “Creek” (*p* < 0.05) (Figure 2H). The thickness of the main vein in “Pawnee” was significantly greater than in the other cultivars, ranging from 16.9% to 21.3% higher (*p* < 0.05); there were no significant differences among the other four cultivars (*p* > 0.05) (Figure 2I). The micrographs below show leaf cross-sections of the five pecan cultivars: “Pawnee”, “Mandan”, “Nacono”, “Caddo”, and “Creek”. The left margin is labeled “D-e,” denoting the lower epidermis; “Pal” on the right indicates the palisade parenchyma, while “Pa” in the upper right marks the upper epidermis. Beneath the palisade layer lies the spongy mesophyll, characterized by loosely arranged cells. The mid-rib vascular bundle is semicircular, with the xylem positioned toward the upper epidermis and the phloem toward the lower epidermis (Figure 2J).

#### 2.2.2. Stem Anatomical Structure

**Figure 3:**
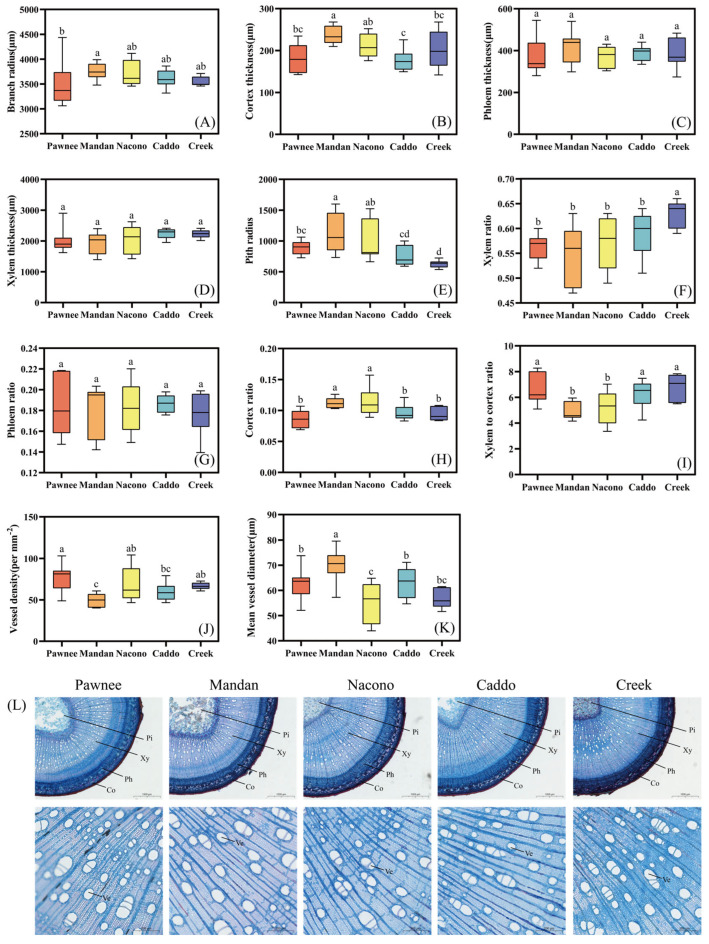
Differences in the anatomical structure of branches of different cultivars of pecan seedlings. Branch radius among different pecan cultivars (**A**). Cortex thickness among different pecan cultivars (**B**). Phloem thickness among different pecan cultivars (**C**). Xylem thickness among different pecan cultivars (**D**). Pith radius among different pecan cultivars (**E**). Xylem ratio among different pecan cultivars (**F**). Phloem ratio among different pecan cultivars (**G**). Cortex ratio among different pecan cultivars (**H**). Xylem to cortex ratio among different pecan cultivars (**I**). Vessel density among different pecan cultivars (**J**). Mean vessel diameter among different pecan cultivars (**K**). Schematic diagram of branch anatomical structure among different pecan cultivars (**L**). Different lowercase letters above the bars indicate the differences in the anatomical structure of branches among different cultivars with *p* < 0.05. Pi, Pith; Xy, Xylem; Ph, Phloem.

The branch radius of “Mandan” was significantly larger than that of “Pawnee” by 7.3% (*p* < 0.05), and except for “Pawnee”, the branch radius of the other four cultivars showed no significant differences (*p* > 0.05) (Figure 3A). Additionally, its cortex was the thickest, being significantly greater than that of “Nacono” by 13.03%, “Creek” by 17.37%, and “Pawnee” by 31.1%, and extremely significantly thicker than that of “Caddo” by 35.83% (*p* < 0.01) (Figure 3B). There was no significant difference in phloem thickness among the five cultivars (*p* > 0.05) (Figure 3C). The mean xylem thickness ranged narrowly from 1949.14 μm (“Mandan”) to 2250.36 μm (“Caddo”), while there was no significant difference among the five cultivars for this index (*p* > 0.05) (Figure 3D). The pith radius of “Creek” was significantly smaller than that of “Pawnee” (*p* < 0.05), extremely smaller than “Mandan” and “Nacono” (*p* < 0.01) (Figure 3E), while its xylem ratio was significantly higher than that of the other cultivars (*p* < 0.05) (Figure 3F). No significant difference in phloem ratio was observed among all cultivars (*p* < 0.05) (Figure 3G). The cortex ratio of “Mandan” and “Nacono” was significantly greater than that of “Pawnee”, “Caddo”, and “Creek” (*p* < 0.05) (Figure 3H), whereas their xylem to cortex ratio exhibited the inverse pattern (*p* < 0.05) (Figure 3I). The vessel density of “Mandan” was significantly lower than that of other cultivars (*p* < 0.05) (Figure 3J), but its diameter was significantly larger than that of the other cultivars (*p* < 0.05) (Figure 3K). The epidermis is the outermost single layer of cells covered by a cuticle. Beneath it, the cortex consists of multiple layers of parenchyma cells. A continuous ring of vascular bundles is arranged around the pith; xylem and phloem are clearly demarcated, with xylem vessels small and densely packed. The centrally located pith is composed of large, loosely arranged cells (Figure 3L).

#### 2.2.3. Leaf Epidermal Micromorphology

**Figure 4:**
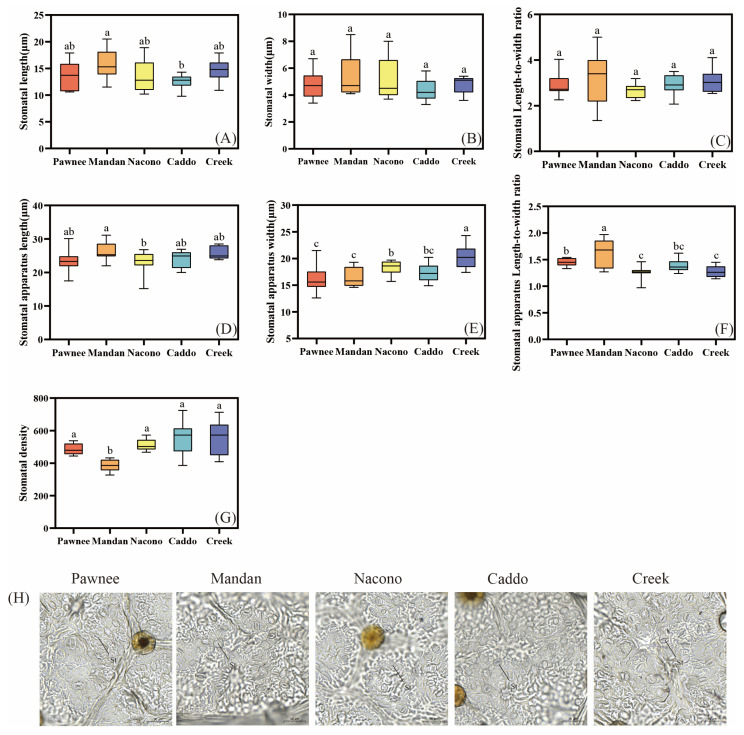
The differences in stomatal structure of leaves of different cultivars of pecan seedlings. Stomatal length among different pecan cultivars (**A**). Stomatal width among different pecan cultivars (**B**). Stomatal length to width among different pecan cultivars (**C**). Stomatal apparatus length among different pecan cultivars (**D**). Stomatal apparatus width among different pecan cultivars (**E**). Stomatal apparatus Length to width among different pecan cultivars (**F**). Stomatal density among different pecan cultivars (**G**). Schematic diagram of leaf epidermal stomatal structures among different pecan cultivars (**H**). Different lowercase letters above the bars indicate the differences in stomatal structure among different cultivars with *p* < 0.05.

The stomatal length of “Mandan” was significantly longer than that of “Caddo” (*p* < 0.05), and stomatal length did not differ significantly among “Pawnee”, “Nacono”, and “Creek” (*p* > 0.05), nor did any of them differ from “Mandan” or “Caddo” (*p* > 0.05) (Figure 4A). No significant differences were observed among the five cultivars in either stomatal width or stomatal length-to-width ratio (*p* > 0.05) (Figure 4B,C). Except for “Mandan”, whose stomatal apparatus length was significantly greater than that of the “Nacono” (*p* < 0.05), no significant differences were observed among the other varieties. (*p* > 0.05) (Figure 4D). The stomatal apparatus width of “Mandan” was significantly wider than that of “Nacono” and “Caddo” (*p* < 0.05), extremely wider than “Pawnee” and “Mandan” (*p* < 0.01) (Figure 4E), and its stomatal apparatus length-to-width ratio was significantly greater than that of the other cultivars (*p* < 0.05) (Figure 4F). The stomatal density of “Mandan” was significantly lower than that of the other cultivars, approximately 20–30% lower (*p* < 0.05) (Figure 4G). Below are scanning electron micrographs of the abaxial epidermis of leaves from five pecan cultivars. Stomata are uniformly distributed; guard cells are reniform, flanked by 2–4 subsidiary cells, and stomatal apertures vary among cultivars. Both guard cell length and stomatal pore diameter differ across genotypes (Figure 4H).

### 2.3. Comparison of Photosynthetic Physiology of Different Cultivars of Pecan at the Seedling Stage

#### 2.3.1. Photosynthetic Pigment Content

**Figure 5:**
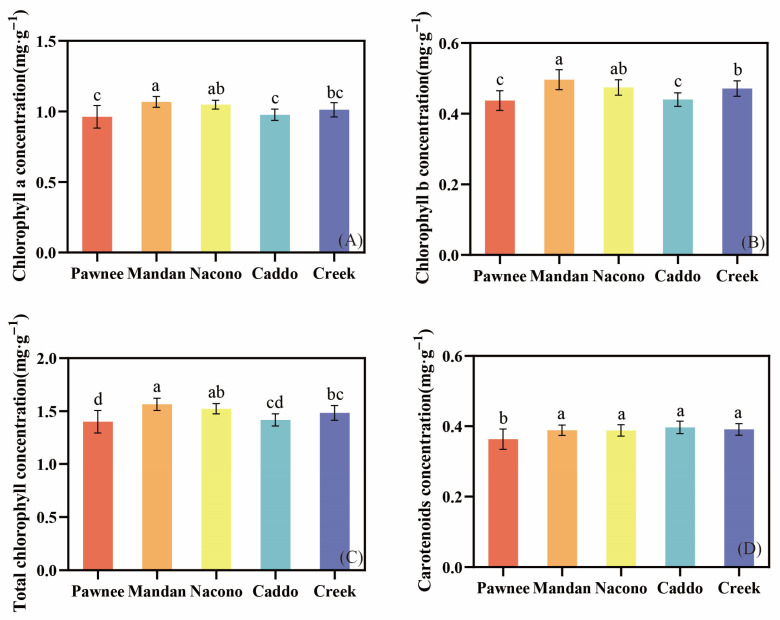
The differences in leaf chlorophyll concentration among different cultivars of pecan seedlings. Chlorophyll a concentration among different pecan cultivars (**A**). Chlorophyll b concentration among different pecan cultivars (**B**). Total chlorophyll concentration among different pecan cultivars (**C**). Carotenoid concentration among different pecan cultivars (**D**). Different lowercase letters above the bars indicate the differences in photosynthetic pigment content among different cultivars with *p* < 0.05.

The total chlorophyll concentration of “Mandan” was the highest (Figure 5C), with both chlorophyll a and chlorophyll b significantly higher than those of the other cultivars (*p* < 0.05) (Figure 5A,B). In contrast, the chlorophyll a and chlorophyll b concentrations of “Pawnee” were significantly lower than those of the other cultivars (*p* < 0.05). The chlorophyll a and chlorophyll b concentrations did not differ significantly among “Mandan”, “Nacono”, and “Creek” (*p* > 0.05) (Figure 5A,B), whereas no significant differences were observed among the other four cultivars (*p* > 0.05) (Figure 5D).

#### 2.3.2. Photosynthetic Parameters

As shown in Table 3, there were no significant differences in intercellular CO_2_ concentration or net photosynthetic rate among the five cultivars (*p* > 0.05). The transpiration rates of “Nacono” and “Caddo” were significantly higher than that of “Pawnee” (*p* < 0.05). The stomatal conductance of “Pawnee” was significantly greater than that of “Mandan” and “Creek” (*p* < 0.05). The water use efficiency of “Pawnee” was significantly higher than that of “Mandan”, “Nacono”, and “Caddo” (*p* < 0.05), while no significant differences in water use efficiency were detected between “Creek” and any other cultivar (*p* > 0.05).

#### 2.3.3. Chlorophyll Fluorescence Parameters

As shown in Table 4, the Fo’ of “Nacono” was significantly higher than that of the other cultivars (*p* < 0.05), extremely significantly higher than that of “Caddo” (*p* < 0.01). The Fm’ of “Caddo” was significantly lower than that of the other cultivars (*p* < 0.05). The Fv/Fo of “Pawnee” was significantly higher than that of the “Nacono”, “Caddo”, and “Creek” (*p* < 0.05). The Fv/Fm of “Pawnee” was significantly higher than that in “Nacono”, “Caddo”, and “Creek” (*p* < 0.05). The PSII of “Caddo” was significantly lower than that of the other cultivars (*p* < 0.05), and no significant differences were observed among the other cultivars (*p* > 0.05). There were no significant differences among the five cultivars in Fo, Fm, qP, or NPQ (*p* > 0.05).

### 2.4. Comprehensive Evaluation of Different Cultivars of Pecan

**Figure 6:**
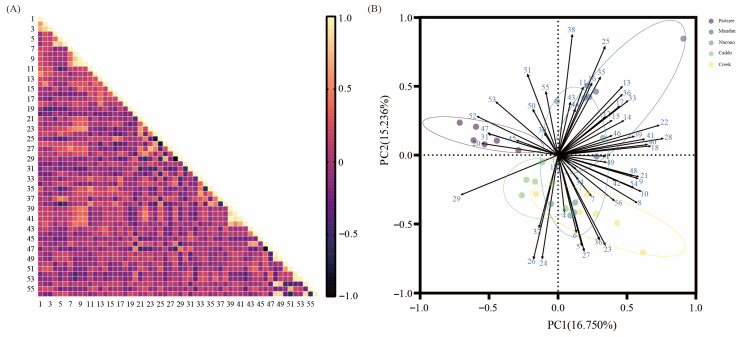
Pearson correlation heat-map of 56 morphological, anatomical, and physiological traits across five pecan cultivars (**A**) (r-values shown; *p* < 0.01 for |r| ≥ 0.70, *p* < 0.05 for 0.50 ≤ |r| < 0.70, *p* > 0.05 for 0.30 ≤ |r| < 0.50). Factor loads of PCA (**B**). 1, plant height; 2, Rootstock diameter; 3, Scion diameter; 4, Leaf length; 5, Leaf weight; 6, Leaf area; 7, Leaf biomass; 8, Stem biomass; 9, Root biomass; 10, Total biomass; 11, Thickness of leaf; 12, Thickness of upper epidermis; 13, Thickness of lower epidermis; 14, Ratio of upper to lower epidermis; 15, Thickness of palisade tissue; 16, Thickness of spongy tissue; 17, Palisade to spongy tissue ratio; 18, Palisade to leaf ratio; 19, Spongy to leaf ratio; 20, Thickness of main vein; 21, Branch radius; 22, Cortex thickness; 23, Phloem thickness; 24, Xylem thickness; 25, Pith radius; 26, Xylem ratio; 27, Phloem ratio; 28, Cortex ratio; 29, Xylem to cortex ratio; 30, Mean vessel diameter; 31, Vessel density; 32, Stomatal density; 33, Stomatal length; 34, Stomatal width; 35, Stomatal length to width ratio; 36, Stomatal apparatus length; 37, Stomatal apparatus width; 38, Stomatal apparatus length to width ratio; 39, Chlorophyll a concentration; 40, Chlorophyll b concentration; 41, Total chlorophyll concentration; 42, Carotenoid concentration; 43, Pn; 44, Gs; 45, Ci; 46, E; 47, WUE; 48, Fo; 49, Fm; 50, Fo′; 51, Fm′; 52, Fv/Fo; 53, Fv/Fm; 54, qP; 55, ΦPSII; 56, NPQ.

Pearson correlation analysis revealed strong positive associations among growth metrics: plant height was tightly linked to both rootstock diameter (r = 0.82, *p* < 0.01) and scion diameter (r = 0.79, *p* < 0.01), while root biomass exhibited a near-perfect correlation with total biomass (r = 0.97, *p* < 0.01). Within leaf anatomy, palisade thickness correlated positively with leaf biomass (r = 0.64, *p* < 0.05), and upper-epidermis thickness with plant height (r = 0.73, *p* < 0.01). Stomatal length and width were significantly coupled (r = 0.58, *p* < 0.05), as were stomatal apparatus length and width (r = 0.71, *p* < 0.01). Chlorophyll variables formed a coherent cluster, with Chl a strongly related to Chl b (r = 0.89, *p* < 0.01) and total chlorophyll to carotenoids (r = 0.86, *p* < 0.01). Photosynthetically, Pn was positively associated with Gs (r = 0.77, *p* < 0.01), and ΦPSII with qP (r = 0.81, *p* < 0.01), underscoring the integrated coordination between morpho-anatomical traits and physiological performance in pecan seedlings (Figure 6A). To study the differences in growth, anatomy, and photosynthesis among grafted pecan seedlings of different cultivars and to evaluate the superiority or inferiority of the cultivars, PCA was conducted on 56 indicators related to growth, anatomy, and photosynthesis. Prior to performing the PCA, all 56 variables were Z-score standardized (mean = 0, standard deviation = 1). The 15 principal components with the largest eigenvalues were extracted. The eigenvalues of the principal components were 9.380, 8.532, 5.292, and so on, with cumulative contribution rates of 16.750%, 15.236%, 9.449%, etc., resulting in a cumulative contribution rate of 93.204% (Table 5). This indicates that the common factors account for 93.204% of the original data variance, thereby retaining the majority of the information without losing important variables (Table 5). Factor loading analysis for the first and second principal components was performed on the *X*-axis and *Y*-axis, respectively (Figure 6B). In the first principal component, indicators with higher load values (>0.5) include Fo, Chla, Chlb, Chl, cortex thickness, cortex ratio, palisade to leaf ratio, branch radius, root biomass, total biomass, stem biomass, and qP, suggesting that these are the primary factors influencing the first principal component. In the second principal component, indicators with larger load values (>0.5) include Fm’, stomatal apparatus length-to-width ratio, pith radius, thickness of spongy tissue, and stomatal length-to-width ratio, identifying these as the main factors contributing to the second principal component. The contribution rates of the principal components were used as weights to calculate the comprehensive scores for grafted pecan seedlings of different cultivars, and the cultivars were ranked accordingly (Table 6). The results showed that the comprehensive scores of the different cultivars were ranked as follows: “Mandan” > “Creek” > “Nacono” > “Caddo” > “Pawnee”.

## 3. Discussion

### 3.1. Differences in the Vegetative Growth of Grafted Pecan Seedlings of Different Cultivars During the Seedling Stage

Different cultivars exhibit morphological differences, which are primarily influenced by their genetic characteristics and growth environment [14,24]. Previous studies have shown that significant variations exist in biological traits, stomatal characteristics, and leaf anatomical structures among different strains of *Eucalyptus* [25]. In this study, no significant differences were observed in plant height, rootstock diameter, and scion diameter among the various pecan cultivars. This may be attributed to the fact that these cultivars adapted to the same growth environment and exhibited similar physiological rhythms and growth synchrony during their growth process [26]. Consistent with previous reports on *Fagus orientalis* and *Acer velutinum* [27], the absence of significant genotypic differences in seedling height in our study likely reflects strong environmental canalization during early ontogeny and/or limited statistical power. The development of leaves is closely linked to photosynthesis and growth adaptability [28]. Environmental factors such as drought [29], waterlogging [30], low temperature [31], and varietal differences [32] all influence leaf morphology and structure. The leaf area of both “Creek” and “Nacono” was significantly larger than that of the other cultivars (*p* < 0.05), conferring a greater light-capture capacity and suggesting that these cultivars may be better adapted to suboptimal light conditions as observed in *Juglans regia fluodianense* [33]. The biomass of different plant organs and their interrelationships can reflect aspects of plant energy distribution and balance [34]. Above-ground and below-ground traits are mutually coordinated in the overall economic spectrum of the plant [35]. The morphological variations of the above-ground portion are closely associated with variations in the root system [36]. *Juglandaceae* plants have well-developed root systems; traits such as root length and root surface area may confer advantages in competition for water and nutrients [37]. “Mandan” and “Creek” possess more developed root systems, which enhance their ability to absorb water and nutrients. As a result, these cultivars demonstrate significantly superior aboveground biomass accumulation compared to others. Some studies suggest that higher biomass accumulation requires a stronger resource allocation capacity for both above-ground and below-ground organs [38]. This indicates that these two cultivars exhibit greater adaptability to environmental conditions. Furthermore, it was observed that the quality index of “Creek” seedlings was significantly better than that of other cultivars, highlighting that, in terms of overall growth indicators, its growth characteristics were the most favorable.

### 3.2. Differences in the Anatomical Structure of Grafted Seedlings of Different Cultivars of Pecan at the Seedling Stage

Under specific environmental conditions, different plants exhibit structural and characteristic differences as an evolutionary adaptation to their respective environments [39]. This study found significant differences in the leaf anatomical structure among various pecan cultivars, which may be closely linked to their stress resistance [40,41,42], and subsequently affect their photosynthetic capacity [43]. In harsh environments, such as drought and high temperatures, thicker leaves may offer more advantages, while in humid environments with low light, thinner leaves may be more beneficial [44]. The leaf thickness of “Pawnee” is significantly greater than that of other cultivars, contributing to its stronger drought resistance. Thick leaves are advantageous for photosynthetic efficiency [18], water retention [45], and stress resistance [46]. In contrast, the leaf thickness of “Mandan” is significantly thinner than that of other cultivars. Thin leaves provide advantages in gas exchange efficiency, temperature regulation, and growth rate. Mohammad M. Arab et al. found that under drought stress, thin leaves exhibited a 60% decline in stomatal conductance but a 25% increase in water use efficiency, indicating that they balance carbon assimilation and water loss through dynamic stomatal regulation. Mesophyll conductance was negatively correlated with leaf thickness; the reduced diffusion resistance in thin leaves helped maintain a high intercellular CO_2_ concentration [47]. The development of palisade tissue is generally associated with efficient photosynthesis and water use efficiency [44]. In this study, “Pawnee” and “Mandan” exhibited thicker palisade tissue, suggesting that these cultivars may possess stronger photosynthetic capacity and drought resistance. Tang et al. discovered that hybrid walnut (*Juglans major* × *Juglans regia*) seedlings sustain photosynthetic capacity by increasing leaf thickness via enhanced palisade and spongy parenchyma development under salt stress [48]. The findings corroborate the hypothesis presented in this study. The spongy tissue of “Mandan” was significantly thinner than that of other cultivars, enabling it to perform photosynthesis more efficiently, reduce water loss, enhance mechanical strength, and lower disease susceptibility, indicating that it is better adapted to adverse environmental conditions. Additionally, the palisade-to-spongy tissue ratio of “Mandan” was significantly higher than that of other cultivars, suggesting that it may improve photosynthetic efficiency by increasing the proportion of palisade tissue. Xiao et al. have suggested that plants with strong drought resistance generally exhibit thicker palisade tissue and a higher palisade-to-spongy tissue ratio [49]. “Mandan” displays a similar structural ratio. The tissue structure compactness of “Mandan” was significantly greater than that of other cultivars, indicating that its leaf structure is more compact and may be better suited to strong light environments [50].

“Mandan” demonstrates outstanding performance in branching structure (diameter, cortex thickness) and vessel diameter, making it well-suited for environments with high mechanical support requirements and stable water supply. Nevertheless, its low vessel density may curtail drought tolerance [51,52]. The pith radius of “Mandan” is the largest, providing ample space for nutrient storage. The xylem of “Caddo” is the thickest, and combined with its highest xylem ratio, this suggests superior water transport efficiency. The xylem thickness of “Creek” is similar to that of “Mandan”, but its pith radius is the smallest, indicating a possible trade-off between storage capacity and enhanced conducting function. The xylem ratio of “Creek” is the highest, highlighting its significant xylem advantage, though its pith storage capacity is relatively weak. Both “Caddo” and “Creek” have highly developed xylems with relatively high xylem ratios, making them well-suited for arid or water-competitive environments. Collectively, both “Caddo” and “Creek” possess highly developed xylem systems suited to water-limited environments, whereas “Mandan” outperforms all cultivars in overall branch structural robustness.

Through the comparative analysis of stomatal parameters across various pecan cultivars, significant differences in stomatal morphology were observed among the cultivars, which may directly impact their photosynthetic efficiency and water use characteristics [53]. Previous studies have shown that small and dense stomata are better adapted to arid environments [54,55]. The stomatal density of “Mandan” is significantly lower than that of the other cultivars, but its stomatal length is the longest, and the aspect ratio of its stomatal apparatus is the highest. These characteristics suggest that the stomatal opening and closing range is large, which may enhance CO_2_ absorption efficiency by increasing stomatal pore size under sufficient light conditions. Similar results were observed for *Juglans regia* [43] and *Populus trichocarp* [56]. “Creek” has relatively large stomatal pores, but excessively high pore conductance could increase the risk of water loss. While “Nacono” exhibits a relatively high stomatal density, its stomatal aspect ratio is the lowest, and its stomatal apparatus width is relatively large. This may result in a slower stomatal closure rate, requiring reliance on non-photochemical quenching to mitigate strong light stress. This is consistent with its slightly lower photosynthetic efficiency but stronger stress resistance.

The phenotypic plasticity of anatomical traits in response to the environment has been documented in many woody species, and pecan is no exception. Previous studies show that parameters such as palisade tissue thickness, stomatal density, and vessel diameter shift significantly with light, water, and temperature: high light or drought typically induce thicker palisade layers and higher stomatal density [57,58], whereas humid or shaded conditions lead to thinner leaves and larger, yet less numerous, stomata [59]. In the study, the thick leaves and high palisade ratio observed in “Pawnee” could confer an even greater drought advantage in dry years or high-radiation sites; conversely, under humid conditions, these same traits may become redundant and reduce photosynthetic efficiency. “Mandan”, with its low stomatal density and long stomata, can curtail transpiration and maintain relatively high WUE in wet years, but extreme drought may elevate diffusive resistance and suppress carbon assimilation.

### 3.3. Differences in Photosynthetic Capacity of Grafted Seedlings of Different Cultivars of Pecan at the Seedling Stage

Different cultivars exhibit variations in photosynthesis, reflected in factors such as chlorophyll content, photosynthetic parameters, and chlorophyll fluorescence characteristics. Using maple, chestnut, wild vine, and beech leaves as study materials, we found that chlorophyll content reflects photosynthetic capacity and is closely linked to plant adaptability to the environment [60,61]. This experiment found significant differences in the accumulation of photosynthetic pigments among different pecan cultivars. “Mandan” had the highest total chlorophyll content and a higher net photosynthetic rate. Both its chlorophyll a and chlorophyll b levels were significantly higher than those of the other cultivars, suggesting that its leaves have a greater ability to capture light energy and may prioritize carbon assimilation through high photosynthetic efficiency [57]. The photosynthetic pigment content of “Nacono” and “Creek” was second to “Mandan”. Notably, the chlorophyll b content of “Nacono” was close to that of “Mandan”, indicating a relatively high antenna pigment ratio in its photosystem II, which may make it more efficient at distributing light energy in low-light environments. In contrast, “Pawnee” had the lowest total chlorophyll content, with both chlorophyll a and chlorophyll b significantly lower than those of the other cultivars. This reflects a relatively weak light energy absorption capacity in its photosynthetic structure, which is consistent with its lower net photosynthetic rate. Although “Pawnee” exhibited significantly higher water use efficiency (WUE) than the other cultivars (*p* < 0.05), it still ranked last in the PCA-based overall assessment. This is because WUE is only one of 56 evaluated traits. “Pawnee” had markedly lower total biomass, root biomass, and stem radius than “Mandan” and “Creek” (Table 2), and its high WUE stemmed mainly from an extremely low transpiration rate rather than from a high photosynthetic rate (Table 3). Thus, “Pawnee” achieves high WUE by drastically reducing stomatal conductance to limit water loss; however, this conservative water use strategy simultaneously constrains total carbon assimilation, resulting in poor biomass accumulation and overall vigor. Consequently, within the integrated evaluation framework, the single advantage of high WUE could not offset its substantial disadvantages in growth and structural robustness, leading to the lowest overall ranking (Table 6).

Chlorophyll fluorescence, as an internal probe of photosynthesis, provides a more accurate reflection of the internal mechanisms of photosynthesis compared to gas exchange parameters, which primarily reflect external characteristics [62]. A comparison of the chlorophyll fluorescence parameters among the leaves of various pecan seedling cultivars revealed that “Pawnee” exhibited significantly higher maximum photochemical efficiency and light energy conversion potential of PSII than the other cultivars. This indicates that the PSII reaction center in “Pawnee” has a strong capacity for light capture and electron transfer, enabling it to maintain high photosynthetic efficiency under high light intensity [63]. “Nacono” demonstrated unique adaptability in the light-adapted state, with both its initial fluorescence and maximum fluorescence being significantly higher than those of other cultivars. Coupled with its higher NPQ, this suggests that “Nacono” may cope with strong light stress by enhancing the non-photochemical quenching mechanism [64]. However, its Fv/Fm ratio was slightly lower than that of “Pawnee”, indicating a trade-off in its photochemical efficiency [65]. The actual photochemical efficiency of “Caddo” was significantly lower than that of the other cultivars, suggesting reduced efficiency in converting light energy into chemical energy. This could be attributed to the activity of the photoreaction center or potential obstruction of the electron transport chain. Despite this, its NPQ was not significantly different from that of the other cultivars, implying that its photoprotection ability remains unaffected [66]. Both “Mandan” and “Creek” performed moderately in most parameters, with their fluorescence values in the light-adapted state showing no significant differences from those of other cultivars. Moreover, neither qP nor NPQ varied among cultivars, suggesting a broadly conserved strategy for partitioning absorbed light between photochemistry and thermal dissipation. All measurements were completed between 9:00 and 11:00 in August 2024 under light conditions confirmed to be optimal for pecan seedlings [1]. Consequently, light, soil moisture, and nutrient levels remained constant in the short term, contributing <5% of the observed variation in Pn and ΦPSII, and were thus insufficient to alter the inter-cultivar difference patterns.

### 3.4. Principal Component Analysis of Seedlings Among Different Pecan Cultivars and Limitations

Prior to performing the PCA, all 56 variables were Z-score standardized (mean = 0, standard deviation = 1). Multicollinearity was then assessed using both the Pearson correlation matrix and the variance inflation factor (VIF). Only four variable pairs exhibited |r| > 0.90, and all VIF values ranged from 1.3 to 6.9—well below the customary threshold of 10. These results indicate the absence of severe multicollinearity, confirming the suitability of the dataset for PCA. Based on the PCA loadings presented in Table 5 and Figure 6B, the first principal component (PC1, 16.75% of variance) was designated as the “vigor–biomass” axis, driven by high positive loadings of root, stem, and leaf biomass, total biomass, branch radius, cortex thickness, and qP (all > 0.50). The second principal component (PC2, 15.24%) represented “structural robustness”, characterized by spongy tissue thickness, pith radius, and the stomatal apparatus length-to-width ratio. The third principal component (PC3, 9.45%) corresponded to “photosynthetic potential”, with dominant contributions from Chla, Chlb, total chlorophyll, Fo, and ΦPSII. Together, these axes provide a biologically interpretable framework for cultivar discrimination at the seedling stage. On PC3, net photosynthetic rate (Pn) has a loading of +0.55, palisade tissue thickness +0.48, and stomatal density −0.42, indicating that Pn covaries positively with palisade thickness and negatively with stomatal density, jointly driving this axis. This result directly demonstrates the coordinated variation of anatomical and photosynthetic variables on the same principal component, providing stronger statistical evidence for their association. This three-dimensional framework can serve as a non-destructive, early-stage screening tool that offers a quantitative solution for shortening the breeding cycle. Future work should expand sample sizes across different climatic zones to validate the stability of the component weights and cultivar rankings. The results showed that the comprehensive scores of the different cultivars were ranked as follows: “Mandan” > “Creek” > “Nacono” > “Caddo” > “Pawnee”. These findings are consistent with the above-discussed results on seedling growth, anatomical traits, and photosynthetic parameters in pecan.

The present study employed six seedlings per cultivar. A priori power analysis (α = 0.05, 1 − β = 0.80). However, biomass and anatomical traits at the seedling stage are highly plastic, and a small sample may underestimate true genetic variation [67]. The series of analyses in this study highlights the importance of integrating multiple statistical strategies in small-sample research. We applied formal outlier tests, complemented parametric with non-parametric methods, and employed resampling techniques to construct a more rigorous and robust inferential framework. Although a limited sample size may reduce statistical power and increase the risk of Type II error, the multi-dimensional validation presented here affords greater confidence in our final conclusions. Because all plant material was evaluated under a single natural environment, the present study was unable to assess genotype × environment interactions, which may limit the broader applicability of our conclusions across different ecological regions. Future multi-site field trials are therefore required for further validation. Future work will expand the sample size (≥15 seedlings per cultivar) and include multi-site trials to validate the robustness of our findings. Grauke et al. reported narrow-sense heritabilities of 0.42 for leaf area and 0.58 for root dry weight in 3-year-old seedling populations, while chlorophyll content reached h^2^ = 0.65—values consistent with early-selection studies in other woody perennials, indicating that seedling traits are under moderate to strong genetic control [68]. Because the present study neither estimated heritability nor conducted multi-year, multi-site validation, we cannot yet claim stability for these traits. Future work should employ half-sib or full-sib family trials coupled with genomic estimated breeding values (gEBV) to further confirm their genetic stability and predictive power for future yield performance. Although phenotypic differences are already apparent at the seedling stage, woody fruit trees typically undergo a pronounced juvenile-to-mature transition, during which leaf anatomy, photosynthetic efficiency, and biomass allocation strategies can be substantially reorganized. Consequently, whether the differences identified here persist into adulthood can only be determined through at least five years of continuous monitoring to distinguish true genetic advantages from transient juvenile effects.

## 4. Materials and Methods

### 4.1. Plant Materials

The plant materials selected include the provincially approved superior cultivars “Pawnee” and “Caddo”, which were bred by the project team at Nanjing Forestry University and authorized by the Jiangsu Provincial Forest Tree Cultivar Review Committee. Additionally, three newly introduced American cultivars—”Mandan”, “Nacono”, and “Creek”—currently undergoing regional testing, were also included. In total, five pecan cultivars were studied, all of which were one-year-old container-grafted seedlings. All cultivars listed here were developed by the USDA Pecan Breeding Center and are interspecific hybrids. “Caddo” (“Brooks” × “Alley”) was released in 1968; “Pawnee” (“Mohawk” × “Starking HG”) in 1984; “Creek” (“Mohawk” × “Starking HG”) in 1996; “Nacono” (“Cheyenne” × “Sioux”) in 2000; and “Mandan” (“BW-1” × “Osage”) in 2009. These cultivars merit exploratory trials across China to provide a scientific basis for commercial-scale pecan cultivation.

### 4.2. Experimental Site

The experimental site is situated at the Pecan Experimental Base of Nanjing Forestry University in Zhangmiao Village, Houbai Town, Jurong City, Jiangsu Province (30°15′50″ N, 119°09′06″ E). This region has a central monsoon climate typical of the northern subtropical zone. The average annual temperature is 15.6 °C, with total annual precipitation of 1018.6 mm. There are 226 days annually with a daily average temperature exceeding 10 °C, and the frost-free period lasts for 229 days. The growing medium was formulated by mixing peat moss, perlite, vermiculite, and coconut coir in a volume ratio of 2:1:1:1. Graft seedlings were cultivated in cylindrical non-woven fabric containers measuring 25 cm in diameter and 30 cm in height (see Table 7).

### 4.3. Experimental Design

This experiment was conducted at the Pecan Experimental Base of Nanjing Forestry University in early April 2024. The rootstock used was a container seedling derived from “Stuart” seeds, and the scion cultivars were “Pawnee”, “Mandan”, “Nacono”, “Caddo”, and “Creek”. A total of 125 one-year-old grafted seedlings (25 per cultivar) were selected for the study. These seedlings were planted outdoors to simulate natural environmental conditions, ensuring consistency in factors such as planting time, environment, and management measures. Six healthy, disease-free plants from each cultivar were randomly selected as standard test plants for growth measurements. The remaining plants were subjected to destructive sampling. Growth observations were made every 10 days from April to November. Plant tissue samples were collected in August for subsequent physiological and biochemical analyses, and biomass measurements were taken in November. Six biological replicates were measured for each cultivar. PCA was applied to conduct a comprehensive evaluation of the five pecan cultivars.

### 4.4. Measurement

#### 4.4.1. Measurement of Growth Indicators

Measurements commenced in April and were conducted every 10 days until the conclusion of the growing season. Biomass was harvested and weighed, while plant height, ground diameter, and ear diameter were measured using a steel tape measure and an electronic digital caliper [69]. Leaf length, width, and area were determined with a portable leaf area scanner (CI-203, CID Bio-Science, Camas, WA, USA) [33]. For each cultivar, six seedlings were randomly selected for measurement, and the process was repeated three times. The average value was recorded until leaf growth ceased.

#### 4.4.2. Observation of Anatomical Structure

Leaves and branches were collected for anatomical analysis, and paraffin sections were prepared. The sections were stained with safranin-green and mounted to create permanent slides for observing the anatomical structure of the leaves [48]. After the sections were prepared, they were examined and recorded using a stereomicroscope with a 20× objective lens. The Slide Viewer software (Version 3.4.0, New York, NY, USA) was utilized to measure and calculate key anatomical parameters, including the thickness of the main vein, leaf thickness, the thickness of the upper and lower epidermis, the ratio of the upper to lower cuticle layer thickness, as well as the thickness of the palisade and spongy tissues. Additionally, the ratio of palisade tissue thickness to spongy tissue thickness, the tissue compactness, and the tissue porosity were observed and recorded.

Branch sections were examined and photographed under the 4× objective lens of the stereomicroscope. Using the Slide Viewer software, a total of nine indicators were analyzed: branch radius, cortex thickness, phloem thickness, xylem thickness, pith radius, xylem ratio, phloem ratio, cortex ratio, and wood bark ratio.

Leaf imprints were obtained using nail polish and were observed and photographed under a 40× objective lens of the stereomicroscope. The Slide Viewer software was then employed to measure stomatal length, stomatal width, stomatal apparatus length, stomatal apparatus width, stomatal area, and stomatal density [70].

#### 4.4.3. Measurement of Photosynthetic Indicators

The CIRAS-2 photosynthesis system (CIRAS-2, PP Systems, Amesbury, UK) was employed to measure key physiological parameters of the leaves, including net photosynthetic rate (Pn), stomatal conductance (Gs), intercellular CO_2_ concentration (Ci), transpiration rate (Tr), and water use efficiency (WUE = Pn/Tr). Chlorophyll content was determined by extraction with pure ethanol, followed by calculation of the concentration [71].

Fluorescence parameters were assessed using a Continuous Excitation Fluorometer (Handy-PEA, Norfolk, UK). The measured fluorescence indices included initial fluorescence yield (Fo), maximum fluorescence yield (Fm), variable fluorescence yield (Fv), photochemical quenching coefficient (qP), and non-photochemical quenching coefficient (NPQ), among others. After 20 min of dark adaptation, the initial fluorescence (Fo) and maximum fluorescence (Fm′) were recorded under dark conditions. Under light-adapted conditions, the actual fluorescence yield (Fo′) and maximum fluorescence yield (Fm′) were measured, with F representing the value of Fo′ [57].

### 4.5. Statistical Analysis

The experimental data were organized using Excel 2019 (Version 2019, Redmond, WA, USA) and SPSS 26.0 (IBM, Armonk, NY, USA). Statistical analysis and significance testing were conducted using data analysis software, with Duncan’s test applied at a significance level of *p* < 0.05. Pearson correlation analysis and the creation of statistical charts were performed using GraphPad Pism9.5.1 (Version 9.5.1, San Diego, CA, USA). Correlation analysis was employed to assess the relationships among physiological growth indicators. Additionally, PCA was performed for each index. The number of principal components was determined based on eigenvalues and cumulative contribution rates, and the principal component scores were calculated from the factor scores. The comprehensive scores for different cultivars were then calculated and ranked based on these principal component scores.

## 5. Conclusions

In this study, we observed differences in vegetative growth, anatomical structure, and photosynthetic capacity among different grafted pecan cultivars during the seedling stage. The diversity in growth among the five grafted pecan seedlings was revealed during this stage. Through principal component analysis and correlation analysis of the various indicators, it was found that Fo, Chla, Chlb, Chl, cortex thickness, cortex ratio, palisade-to-leaf ratio, branch radius, root biomass, total biomass, stem biomass, and qP were the most important key indicators for the comprehensive quality evaluation of pecan graft seedlings. Among the cultivars studied, “Mandan” exhibited the best seedling quality. “Mandan” possesses a well-developed root system ensuring efficient water and nutrient acquisition, thin leaves that enhance gas-exchange rates and thermal regulation, and comparatively thick palisade tissue coupled with elevated total chlorophyll concentration, collectively conferring superior photosynthetic performance and drought tolerance. The remaining cultivars were ranked as follows: “Creek”, “Nacono”, “Caddo”, and “Pawnee”. The “Mandan” cultivar is therefore recommended as a resource for future promotion and production. Performance was optimal only under the conditions of the present trial; widespread deployment, therefore, requires multi-location and multi-year validation. This study provides a theoretical foundation for the production, management, and breeding of pecan.

## Figures and Tables

**Table 1:** Differences in leaf area among different cultivars of pecan seedlings. Different lowercase letters represent the differences in leaf-related indices among different cultivars with *p* < 0.05.

Cultivars	Leaf Length (cm)	Leaf Width (cm)	Leaf Area (mm^2^)	Length/Width Ratio
Pawnee	71.39 ± 14.33 b	37.94 ± 5.09 c	2057.02 ± 543.61 b	1.90 ± 0.40 a
Mandan	76.04 ± 15.67 ab	37.59 ± 7.50 c	2258.92 ± 734.31 ab	2.03 ± 0.31 a
Nacono	70.70 ± 23.35 b	49.09 ± 4.80 a	2702.37 ± 991.47 a	1.43 ± 0.39 b
Caddo	67.29 ± 12.86 b	41.51 ± 6.38 bc	2136.19 ± 602.86 b	1.63 ± 0.26 b
Creek	87.67 ± 17.95 a	44.36 ± 7.44 b	2776.31 ± 692.52 a	1.99 ± 0.36 a

**Table 2:** Differences in biomass and seedling quality index of pecan seedlings of different cultivars. Different lowercase letters indicate the differences in biomass and seedling quality index among different cultivars with *p* < 0.05.

Cultivars	Leaf Biomass (g)	Stem Biomass (g)	Root Biomass (g)	Total Biomass (g)	Shoot/Root Ratio	Graftling Quality Index
Pawnee	24.85 ± 4.08 a	26.66 ± 7.44 b	99.98 ± 22.71 b	151.50 ± 31.54 b	1.92 ± 0.13 a	27.10 ± 4.61 b
Mandan	28.90 ± 13.23 a	41.84 ± 12.88 ab	142.98 ± 32.45 a	213.72 ± 50.58 a	2.13 ± 0.37 a	30.41 ± 5.36 b
Nacono	31.27 ± 6.62 a	32.47 ± 9.89 ab	109.17 ± 25.74 ab	172.90 ± 34.78 ab	1.80 ± 0.20 a	27.36 ± 3.66 b
Caddo	30.59 ± 8.30 a	36.21 ± 9.54 ab	111.20 ± 23.20 ab	178.00 ± 39.14 ab	1.66 ± 0.08 a	27.06 ± 5.52 b
Creek	27.47 ± 8.65 a	48.41 ± 19.57 a	139.55 ± 38.38 a	215.43 ± 60.21 a	1.91 ± 0.37 a	39.47 ± 12.79 a

**Table 3:** Differences in photosynthetic parameters of leaves of different cultivars of pecan seedlings. Different lowercase letters indicate the differences in photosynthetic parameters among different cultivars with *p* < 0.05. Ci, Intercellular CO_2_ concentration; E, Transpiration rate; Gs, Stomatal conductance; Pn, Net photosynthetic rate; WUE, Water use efficiency.

Cultivars	Ci (ppm)	E (mmol·m^−2^·s^−1^)	Gs (mmol·m^−2^·s^−1^)	Pn (μmol·m^−2^·s^−1^)	WUE (%)
Pawnee	156.50 ± 28.37 a	2.60 ± 0.80 b	281.50 ± 53.03 a	11.63 ± 1.85 a	5.25 ± 1.69 a
Mandan	176.00 ± 29.58 a	3.37 ± 0.58 ab	241.67 ± 13.92 b	12.45 ± 1.26 a	3.77 ± 0.65 b
Nacono	182.33 ± 40.72 a	3.50 ± 0.60 a	251.00 ± 16.61 ab	11.15 ± 1.37 a	3.22 ± 0.37 b
Caddo	166.67 ± 49.34 a	3.45 ± 0.59 a	250.50 ± 16.50 ab	11.90 ± 1.37 a	3.50 ± 0.48 b
Creek	159.00 ± 17.99 a	2.80 ± 0.67 ab	236.83 ± 14.47 b	11.28 ± 1.21 a	4.16 ± 0.72 ab

**Table 4:** Differences in chlorophyll fluorescence parameters of leaves of different cultivars of pecan seedlings. Different lowercase letters represent the differences in chlorophyll fluorescence parameters among different cultivars with *p* < 0.05. Fo, Minimum fluorescence (dark-adapted); Fm, Maximum fluorescence (dark-adapted); Fo′, Minimum fluorescence (light-adapted); Fm′, Maximum fluorescence (light-adapted); Fv/Fo, Potential activity of Photosystem II; Fv/Fm, Maximum photochemical quantum yield of photosystem II; qP, Photochemical quenching coefficient; ΦPSII, Actual photochemical efficiency of photosystem II; NPQ, Non-photochemical quenching coefficient.

Cultivars	Fo	Fm	Fo′	Fm′	Fv/Fo	Fv/Fm	qP	ΦPSII	NPQ
Pawnee	281.44 ± 40.84 a	1633.67 ± 196.80 a	298.22 ± 11.03 bc	1110.56 ± 33.39 abc	4.82 ± 0.18 a	0.83 ± 0.01 a	0.98 ± 0.05 a	0.73 ± 0.01 a	0.47 ± 0.18 a
Mandan	300.89 ± 41.49 a	1678.67 ± 205.92 a	307.11 ± 16.81 ab	1130.56 ± 81.14 ab	4.59 ± 0.23 ab	0.82 ± 0.01 ab	1.00 ± 0.05 a	0.73 ± 0.01 a	0.49 ± 0.21 a
Nacono	302.67 ± 24.41 a	1644.33 ± 205.33 a	320.89 ± 15.82 a	1167.44 ± 94.84 a	4.43 ± 0.43 b	0.81 ± 0.02 bc	0.98 ± 0.04 a	0.73 ± 0.02 a	0.43 ± 0.28 a
Caddo	283.78 ± 42.34 a	1512.11 ± 211.50 a	308.56 ± 18.61 ab	1036.33 ± 80.03 c	4.34 ± 0.30 b	0.81 ± 0.01 c	0.98 ± 0.07 a	0.70 ± 0.02 b	0.48 ± 0.29 a
Creek	279.33 ± 37.77 a	1596.47 ± 201.89 a	289.11 ± 10.33 c	1056.44 ± 83.43 bc	4.44 ± 0.23 b	0.82 ± 0.01 bc	0.99 ± 0.05 a	0.73 ± 0.02 a	0.44 ± 0.24 a

**Table 5:** The rate of eigenvalue, contribution, cumulative contribution, and factor weight in principal components.

Principal Component	Eigenvalue	Variance Contribution Rate/%	Cumulative Contribution Rate/%	Factor Weight
F1	9.380	16.750	16.750	0.180
F2	8.532	15.236	31.986	0.163
F3	5.292	9.449	41.435	0.101
F4	4.712	8.414	49.849	0.090
F5	4.207	7.513	57.363	0.081
F6	3.753	6.702	64.064	0.072
F7	3.378	6.032	70.096	0.065
F8	2.461	4.394	74.490	0.047
F9	2.395	4.277	78.767	0.046
F10	1.779	3.177	81.945	0.034
F11	1.484	2.650	84.595	0.028
F12	1.347	2.405	86.999	0.026
F13	1.248	2.228	89.227	0.024
F14	1.147	2.049	91.276	0.022
F15	1.080	1.928	93.204	0.021

**Table 6:** Scores of different cultivars in the principal component and comprehensive evaluation.

Cultivars	Scores	Ranking
Pawnee	−3.848	5
Mandan	2.667	1
Nacono	−0.536	3
Caddo	−0.566	4
Creek	2.283	2

**Table 7:** Values of soil indicators at the experimental site. PH, potential of Hydrogen; SOC—Soil, Organic Carbon; TN, Total Nitrogen; TP, Total Phosphorus; TK, Total Potassium; Olsen-P, Olsen-extractable Phosphorus; Ex-K: Exchangeable Potassium; Ex-Ca: Exchangeable Calcium.

Soil Indicators	PH	SOC	TN	TP	TK	Olsen-P	Ex-K	Ex-Ca
Values	6.92	102.4 g·kg^−1^	9.5 g·kg^−1^	3.3 g·kg^−1^	5.13 g·kg^−1^	8.02 mg·kg^−1^	86.74 mg·kg^−1^	800.56 mg·kg^−1^

## Data Availability

This study did not report any data.
